# Nurse‐led task‐shifting strategies to substitute for mental health specialists in primary care: A systematic review

**DOI:** 10.1111/ijn.13046

**Published:** 2022-03-13

**Authors:** Gading Ekapuja Aurizki, Ian Wilson

**Affiliations:** ^1^ Faculty of Nursing Universitas Airlangga Surabaya East Java Indonesia; ^2^ Advanced Leadership for Professional Practice (Nursing) Programme The University of Manchester Manchester UK; ^3^ Division of Nursing, Midwifery and Social Work, School of Health Sciences The University of Manchester Manchester UK

**Keywords:** mental health, nurses, primary care, roles, task‐sharing, task‐shifting

## Abstract

**Aim:**

The study aimed to synthesize evidence comparing task‐shifting interventions led by general practice nurses and mental health specialists in improving mental health outcomes of adults in primary care.

**Design:**

This study used a systematic review of randomized controlled trials.

**Data Sources:**

Articles from the databases CINAHL, MEDLINE, APA PsycInfo, PubMed, EMBASE, Cochrane EBM Reviews, Web of Science Core Collection, and ProQuest Dissertation and Thesis published between 2000 and 2020 were included.

**Review Methods:**

The review was arranged based on the Cochrane Collaboration guidelines and reported using the Preferred Reporting Items for Systematic Reviews and Meta‐Analyses (PRISMA).

**Results:**

Twelve articles met the eligibility criteria. Eight studies revealed that nurse‐led intervention was significantly superior to its comparator. The review identified three major themes: training and supervision, single and collaborative care and psychosocial treatments.

**Conclusion:**

Nurses could be temporarily employed to provide mental health services in the absence of mental health specialists as long as appropriate training and supervision was provided. This finding should be interpreted with caution due to the high risk of bias in the studies reviewed and the limited generalisability of their findings.

## Summary statement

What is already known about this topic?

- The shortage of psychiatrists and other mental health professionals has been causing treatment gaps in mental health care, not only in developing countries but also in developed countries.
- Task‐shifting or task‐sharing is one of the most promising strategies in addressing the treatment gap, but the involvement of nurses in this strategy is barely studied.

What this paper adds?

- This paper explores the effect of task‐shifting and task‐sharing interventions led by general practice nurses for adults with mental disorders in primary care and found that nurses could deliver psychosocial interventions normally delivered by mental health specialists with at least the same quality provided they received proper training and supervision.
- This paper presented a theoretical framework of task‐shifting.

The implications of this paper:

- In the absence of mental health services in primary care, general practice nurses could be temporarily employed to fill the vacancies.
- However, appropriate training and supervision are required before the programme is started to make sure that the nurses are adequately prepared to undertake the new roles in practice.
- The implementation of task‐shifting and task‐sharing requires a well‐planned and gradual implementation strategy to avoid burnout and workplace‐related distress in the target health workers.

## 1 INTRODUCTION

The shortage of psychiatrists and other mental health professionals has been causing treatment gaps in mental health care, not only in developing countries but also developed countries (Bruckner et al., 2011; Butryn et al., 2017). This problem becomes a global concern as mental disorders are among the leading factors for the global burden of disease (Hay et al., 2017). Many people with mental health problems, particularly mood and anxiety disorders, did not get proper treatments they need (Thornicroft, 2007). The treatment gap reached 63% in high‐income countries and even higher in upper‐middle‐income (78%) and lower‐middle‐income countries (86.3%) (Alonso et al., 2018; Evans‐Lacko et al., 2018).

Despite the shortage, the conventional model of mental health‐care deliveries (i.e. one‐to‐one treatment session, at a health‐care facility and by a highly qualified provider) is still widely implemented (Kazdin, 2017; Patel et al., 2011). This model is insufficient to reach more people to get treated as most mental health specialists are usually concentrated in urban areas, and many people are unable to go to health‐care facilities due to various reasons (Kazdin, 2017). There are some strategies proposed to address this issue, namely, increasing the production of mental health professionals, developing psychosocial interventions, utilizing innovative technological platform and involving nonspecialist health workers (NSHWs) (Kazdin, 2017; Patel et al., 2010; Rebello et al., 2014). The involvement of NSHWs in mental health‐care delivery, often called as task‐shifting or task‐sharing, is arguably the most prominent strategy.

Task‐shifting, by definition, is ‘a process whereby specific tasks are moved, where appropriate, to health workers with shorter training and fewer qualifications’ (p.7) (WHO, 2008). Task‐shifting is often used interchangeably with task‐sharing, but the latter term is implemented in more collaborative ways (Orkin et al., 2021). Despite the differences, the basic idea of both approaches is to utilize the available NSHWs to provide mental health‐care services in the absence of mental health specialists. For convenience purpose, this article uses ‘task‐shifting’ to refer to both approaches, unless otherwise specified.

Many articles have discussed the task‐shifting strategy; mental health is the sixth most discussed cluster in task‐shifting research (Benton et al., 2020). Between 2013 and 2020, 11 systematic reviews discussed the implementation of task‐shifting in mental health context (Dham et al., 2017; Ekers et al., 2013; Galvin & Byansi, 2020; Halcomb et al., 2018; Ho et al., 2016; Hoeft et al., 2018; Padmanathan & De Silva, 2013; Shahmalak et al., 2019; van Ginneken et al., 2013; van Straten et al., 2015; Verhey et al., 2020). These reviews have various concerns in terms of settings, mental disorders, treatment providers involved, methodologies included and interventions provided. However, only two systematic reviews focused on nurses as the leading providers of task‐shifting (Ekers et al., 2013; Halcomb et al., 2018). One review summarized mental health interventions led by general practice nurses, hereinafter referred to as the nurses, in primary care without sufficiently exploring the task‐shifting outcomes (Halcomb et al., 2018). Meanwhile, the other review focused on one aspect of task‐sharing but was not comprehensive enough to cover the task‐shifting counterpart (Ekers et al., 2013).

The involvement of nurses in task‐shifting interventions is arguably underestimated. Some authors did not recognize their professional roles and often misleadingly generalized them as community health workers (Afolabi et al., 2019; Gureje et al., 2019; Ola & Atilola, 2019). Moreover, some reviews did not distinguish the role of the nurses from doctors and social workers and referred to all of them as NSHWs (van Ginneken et al., 2013; Verhey et al., 2020). Accordingly, a systematic review that focuses on the involvement of nurses in substituting mental health specialists is needed to complete the body of evidence regarding task‐shifting research.

## 2 REVIEW METHODS

### 2.1 Aims

This review aimed to synthesize evidence concerning task‐shifting interventions led by general practice nurses compared with usual or specialist care in improving the mental health outcomes of adult patients in primary care.

### 2.2 Design

This study used a systematic review design guided by the Cochrane Handbook guidelines (Higgins et al., 2019) and reported using the Preferred Reporting Items for Systematic Reviews and Meta‐Analyses (PRISMA) statement (Moher et al., 2009). The first author conducted the literature search and analysis, while the second author reviewed the abstracts of the final studies included and did not express any disagreement regarding the eligibility criteria.

### 2.3 Eligibility criteria

The eligibility criteria were determined based on the PICOS format, namely Participants and Places, Intervention, Comparisons, Outcomes and Study design, as follows:

#### 2.3.1 Participants and places

This review included studies where the participants were primarily diagnosed with mental health problems. However, studies focused on alcohol and substance use or neurological disorders were excluded. The participants must be adults, 18 years old or above. Regarding the settings, the review included only studies conducted in primary care or community settings and excluded studies conducted in hospitals and hospital‐based outpatient clinics. To avoid distraction from physical‐based usual care, this review excluded studies which limited the participants only to those who have physical‐related conditions such as pregnant women, postpartum mothers and patients with chronic illnesses or disabilities.

#### 2.3.2 Intervention

The studies must involve various task‐shifting interventions, among others, employing NSHWs in mental health interventions, collaborative and stepped care, training and supervision, transdiagnostic or staged interventions, and digital innovations (Patel et al., 2018; Raviola et al., 2019).

#### 2.3.3 Comparisons

This review specified the task‐shifting comparison between nurses who have little experience or no formal qualification in mental health field and mental health specialists (e.g. psychiatrist, psychologist or mental health/psychiatric nurse) or general practitioners (GPs). The studies must involve at least one intervention arm provided by nurses and at least one comparison arm provided by mental health specialists or GPs.

#### 2.3.4 Outcomes

The included studies should report at least one mental health outcome with a valid instrument. Studies that did not report the mental health outcome or used unclear instrument were excluded.

#### 2.3.5 Study design

This review only included studies using a randomized controlled trial (RCT) design published between 2000 and 2020 with accessible full‐text in English. Any non‐randomized study or publication before 2000 or where the full‐text was not available or reported in any language other than English was deemed ineligible.

### 2.4 Literature search strategy

The reviewers generated the articles from eight databases between 2 and 3 July 2020, namely, CINAHL, EMBASE, MEDLINE, APA PsycInfo, Cochrane Database of Systematic Reviews and Web of Science Core Collection, as well as Proquest Dissertation and Thesis for grey literature. Additional articles were searched through reference list tracking. The search terms were determined based on four concepts: *primary health care, nurse‐led task‐shifting, mental disorder* and *RCT*. The Boolean search formulation and detailed search terms of these concepts are described in supporting information S1.

### 2.5 Study selection

**FIGURE 1:**
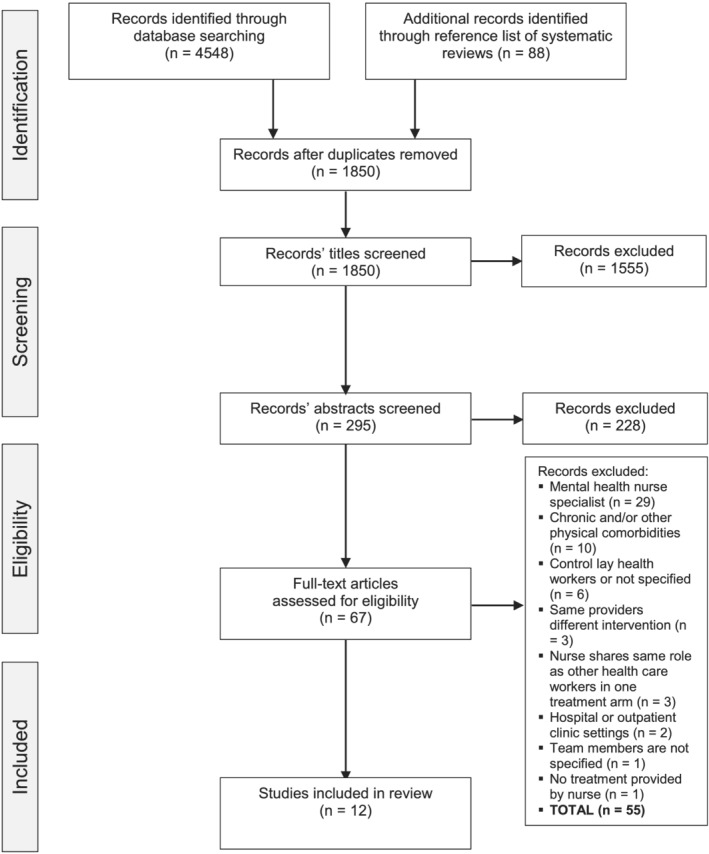
The Preferred Reporting Items for Systematic Reviews and Meta‐Analyses (PRISMA) flow diagram

Initial searching obtained 4548 articles. Additional articles were generated through reference lists searching and grey literature, totalling 88 articles. A total of 1850 articles remained following the removal of duplicates. The title and abstract screening excluded 1555 and 228 articles, respectively, leaving 67 remaining articles for full‐text screening. From these, 12 articles met the eligibility criteria, 55 articles were excluded because the studies focused on mental health nurse specialists (*n* = 29), on participants with chronic or physical comorbidities (*n* = 10), the comparisons were lay health workers or not specified (*n* = 6), the nurses shared the same role with other health workers (*n* = 3), the studies focused on the differences between the interventions instead of the providers (*n* = 3), the settings were in hospitals or outpatient clinics (*n* = 2), the team members were not specified (*n* = 1) and no treatment arm was provided by nurses (*n* = 1). The PRISMA flow diagram summarizes the study selection process (see Figure 1).

### 2.6 Data extraction and analysis

Data were extracted and inputted to Covidence (https://app.covidence.org/). As the studies included have a high degree of heterogeneity, including various intervention durations and outcome measurements (see Appendix Table A1), statistical synthesis or meta‐analysis of data was not appropriate. Therefore, this review used thematic analysis to summarize the most important issues and themes found in the body of literature (Mays et al., 2005).

### 2.7 Quality appraisal

This review used the Cochrane tool for assessing the risk of bias (RoB) in randomized trials (Sterne et al., 2019), which consists of five domains: randomization process, deviations from intended interventions, missing outcome data, measurement of the outcome and the selection of the reported result. The results of RoB assesments were summarized in Table 1.

**TABLE 1:** The Cochrane risk of bias assessment

Study	R	D	Mi	Me	S	Overall risk of bias
Aragonès et al. (2012)*	+	?	?	+	+	Some concerns
Buszewicz et al. (2016)	+	?	?	?	+	Some concerns
Casañas et al. (2012)*	+	+	+	−	+	High
Dobscha et al. (2006)	?	−	?	+	+	High
Ejeby et al. (2014)*	+	?	?	−	+	High
Fortney et al. (2013)*	?	−	?	+	+	High
Hunkeler et al. (2000)*	−	−	+	−	−	High
Malakouti et al. (2015)*	?	−	−	+	−	High
Mynors‐Wallis et al. (2000)	+	−	−	+	−	High
Rost et al. (2001)*	?	−	?	+	+	High
Rost et al. (2002)*	?	+	+	?	+	Some concerns
Zimmermann et al. (2016)	−	−	+	−	+	High
Low risk of bias	5	2	4	6	9	0
Some concerns	5	3	6	2	0	3
High risk of bias	2	7	2	4	3	9

*Note*: R, bias arising from the randomization process; D, bias due to deviations from intended interventions; Mi, bias due to missing outcome data; Me, bias in measurement of the outcome; S, bias in selection of the reported result; O, overall risk of bias; +, low risk of bias; ?, some concerns; −, high risk of bias.

* *p* < 0.05.

## 3 RESULTS

### 3.1 Study characteristics

The studies included a total of 3755 participants randomized into the treatment groups. Most participants were female (*n* = 2527; 67.3%). All but one study was conducted in high‐income countries (*n* = 11; 91.6%). Overall, the studies were carried out in 159 centres, 70 of which were located in urban areas (44.0%). Eleven studies (91.6%) involved patients with depression, four of which were specified as major or chronic depression. Only one study focused on severe mental illness: bipolar or schizophrenia spectrum disorders. The baseline duration of interventions across the studies spanned from 1 week to 12 months. The characteristics of the 12 included studies, and the outcome measurements were described in Table 2.

**TABLE 2:** The characteristics of the included studies and risk of bias assessment

Study	Country (centres)	Baseline participants	Treatment arms and the providers	Main findings	Risk of bias
Aragonès et al. (2012)*	Spain (20 centres)	388 people with depression (79.3% female)	1) Multicomponent programme led by nurse case manager, in collaboration with GP and psychiatrist practices (shared consultation). Duration: 1 week after inclusion then monthly until remission. 2) Usual care by GP	Multicomponent care led by a nurse was more effective than usual care led by a GP in improving depression severity at 3 and 6 months (both *p* = 0.009). Besides, the response and resmission were significantly higher in the nurse‐led arm.	Some concerns
Buszewicz et al. (2016)	UK (42 centres)	558 people with chronic depression (74.9% female)	1) Proactive care led by nurse case manager, in collaboration with GP (shared consultation). Duration: 10 appointments offered after 1 month, 2 months, and every 3 months for 2 years. 2) Usual care by GP	The BDI‐II outcome of patients in proactive care group led by nurse and usual care group led by GP was not statistically different (*p* = 0.125).	Some concerns
Casañas et al. (2012)*	Spain (12 centres)	231 people with Major depression (89.2% female)	1) Psychoeducational interventions by two nurses. Duration: 12 weeks. 2) Usual care by GP and nurse	Psychoeducational interventions by nurses could produce higher remission rate of BDI than the usual care at 3‐ (*p* = 0.005), 6‐ and 9‐month (both *p* = 0.014) follow‐ups.	High
Dobscha et al. (2006)	USA (5 centres)	375 people with depression (6.9% female)	1) Depression decision support team led by nurse care manager, in collaboration with a psychiatrist (shared consultation). Duration: 1–3 weeks. 2) Usual care by GP plus nurses and/or physician assistant	The HSCL‐20 score at 12 months was not significantly different in depression decision support and usual care groups (*p* = 0.49), but the nurse‐led group had greater satisfaction (*p* = 0.002), improved care process (*p* = 0.003) and follow‐up action (*p* < 0.001).	High
Ejeby et al. (2014)*	Sweden (1 centre)	245 people with common mental disorders (80.8% female)	1) Multimodal intervention (MMI) by nurse. Duration: 6 weeks. 2) Cognitive behavioural therapy (CBT) by psychologist. Duration: 12 weeks. 2) Usual care by GP	MMI led by nurses produced a higher mean improvement on SF‐36 Mental Health than CBT led by psychologist (*p* = 0.02) and usual care led by GP (*p* = 0.001) at 2‐week and 12‐month follow‐ups.	High
Fortney et al. (2013)*	USA (5 centres)	364 people with depression (81.6% female)	1) Collaborative telemedicine team led by nurse care manager, in collaboration to pharmacist, psychologist or psychiatrist (stepped care referral). Duration: 12 months. 2) Practice‐based intervention by nurse or GP. Duration: 12 months.	The telemedicine group led by a nurse had significantly higher mean improvement, response and remission rates of HSCL‐20 than practice‐based group in 6‐, 12‐ and 18‐months follow‐ups (*p* < 0.001).	High
Hunkeler et al. (2000)*	USA (2 centres)	302 people with major depressive disorder or dysthymia (45.7% female)	1) Telehealth intervention led by nurse, in collaboration with GP (shared consultation) plus peer support. Duration: 4 months. 2) Usual care by GP	The ≥50% improvement of depression was significantly higher in nurse telehealth group compared with usual care at 6 weeks (*p* = 0.01) and 6 months (*p* = 0.003).	High
Malakouti et al. (2015)*	Iran (4 centres)	176 people with severe mental illness (36.4% female)	1) Home visits led by nurse, in collaboration with a psychiatrist (referral and counter‐referral). Duration: every 2 weeks in the first 3 months and then once every month until 12 months. 2) Home visits led by GP. Duration: Every 2 weeks in the first 3 months and then once every month until 12 months. 2) Usual care by referral to a psychiatrist	The Young Mania Rating Scale in nurse‐led home visits was significantly lower than in the GP‐led home visits and usual care (*p* = 0.03).	High
Mynors‐Wallis et al. (2000)	UK (24 centres)	151 people with major depression (76.8% female)	1) Problem‐solving therapy led by nurse. Duration: 12 weeks. 2) Problem‐solving therapy led by GP. Duration: 12 weeks. 3) Medication 4) Combination of medication and problem‐solving therapy	Despite the improvements of BDI or HDRS in all treatment groups, the differences among groups were not significant (all *p* > 0.1).	High
Rost et al. (2001)*	USA (12 centres)	479 people with depression (83.9% female)	1) Collaborative care led by nurse, involving GP and administrative staff (collaboration nonhierarchical). Duration: baseline 6 months and continuing intervention from 7 to 24 months. 2) Usual care by GP, plus nurse and administrative staff	The nurse‐led collaborative care significantly improved the mCES‐D score compared with the usual care (*p* = 0.04).	High
Rost et al. (2002)*	USA (12 centres)	211 people with depression (83.9% female)	1) Nurse care manager, added to GP practice (shared consultation), supported by office staff. Duration: 3 months. 2) Usual care by GP	The nurse‐led enhanced care showed increased remission (i.e. mCES‐D score <16) compared with usual care across times (*p* = 0.02).	Some concerns
Zimmermann et al. (2016)	Germany (20 centres)	325 people with anxiety, depression, somatic symptoms (66.8% female)	1) Nurse‐led self‐management therapy, in collaboration with GP (shared consultation). Duration: 12 months. 2) Usual care by GP	The self‐management therapy led by nurses significantly improved the patients' self‐efficacy compared with the the control group (*p* = 0.004), but the PHQ‐D outcomes were not significantly different between groups (*p* > 0.05).	High

Abbreviations: BDI, Beck Depression Inventory; CBT, cognitive behaviour therapy; GP, general practitioner; HDRS, Hamilton Depression Rating Scale; mCES‐D, modified Centre for Epidemiological Studies Depression; PHQ‐D, Patient Health Questionnaire‐Depression.

* *p* < 0.05.

### 3.2 Outcomes

There are three types of outcomes reported in the included studies: mental health (*n* = 12), patient satisfaction (*n* = 5) and cost‐effectiveness (*n* = 1).

#### 3.2.1 Mental health

Most studies (*n* = 8) suggested that nurse‐led interventions were significantly superior to the comparators in improving the participant mental health outcomes. In Aragonès et al. (2012), the multicomponent care led by a nurse was significantly more effective than usual care delivered by a GP in improving depression severity at 3 and 6 months (both *p* = 0.009). The response and remission rates were significantly higher in the nurse‐led group at 3‐, 6‐ and 12‐month follow‐ups (*p* < 0.05). In Casañas et al. (2012), the remission rates were also significantly higher in the nurse‐led group at 3 (*p* = 0.005), 6 and 9 months (both *p* = 0.014). Ejeby et al. (2014) revealed that multimodal interventions delivered by a nurse had a higher mean improvement on SF‐36 Mental Health than cognitive behaviour therapy (CBT) delivered by a psychologist (*p* = 0.02) and usual care by GP (*p* = 0.001) at 2 weeks and 12 months.

Fortney et al.'s (2013) telemedicine group led by a nurse had significantly higher mean improvement, response and remission rates of Hopkins Symptom Checklist‐20 (HSCL‐20) than practice‐based group in 6‐, 12‐ and 18‐month follow‐ups (*p* < 0.001). In Hunkeler et al. (2000), the response rates were significantly higher in the nurse telehealth group compared with usual care at 6 weeks (*p* = 0.01) and 6 months (*p* = 0.003). However, based on the Beck Depression Inventory (BDI), the difference of response rates between the groups was not significant at 6 weeks (*p* = 0.28) but was significant at 6 months (*p* = 0.05). The Young Mania Rating Scale in Malakouti et al.'s (2015) nurse‐led home visits was significantly lower than in the GP‐led home visits and usual care (*p* = 0.03). In Rost et al. (2001), the improvement of modified Centre for Epidemiological Studies Depression (mCES‐D) scores in the nurse‐led intervention group was statistically significantly higher than usual care (*p* = 0.04). Meanwhile, Rost et al.'s (2002) nurse‐led enhanced care showed an increased remission compared with usual care across times (*p* = 0.02).

In four studies, the nurse‐led intervention groups were not statistically significantly different from the comparators. Buszewicz et al. (2016) revealed that the BDI‐II between the treatment groups was not statistically different (*p* = 0.125). In Dobscha et al. (2006), the HSCL‐20 score at 12 months was not significantly different in both groups (*p* = 0.49). Mynors‐Wallis et al. (2000) concluded that there were improvements in BDI or Hamilton Depression Rating Scale (HDRS) scores in all treatment groups, but the differences among groups were not significant (all *p* > 0.1). Meanwhile, in Zimmermann et al. (2016), the improvement of self‐efficacy in nurse‐led intervention groups was more significant than the control group (*p* = 0.004), but the Patient Health Questionnaire‐Depression (PHQ‐D) outcomes were not significantly different between groups (*p* > 0.05).

#### 3.2.2 Patient satisfaction

Five studies assessed patient satisfaction. Two studies used validated instruments, that is, the Consumer Assessment of Healthcare Providers and Systems (CAHPS) (Fortney et al., 2013) and the eight‐item version of Attkisson and Zwick's Client Questionnaire Satisfaction (CQS‐8) (Malakouti et al., 2015). Three studies asked each participant about their satisfaction with 5‐point Likert scale response (Dobscha et al., 2006; Hunkeler et al., 2000; Rost et al., 2001). All studies claimed patient satisfaction in the nurse‐led group were significantly higher than in the comparison group. However, Fortney et al.'s (2013) satisfaction claim was based on significance at 0.10 level (*p* = 0.08), different than the common 0.05 level.

#### 3.2.3 Cost‐effectiveness

Only one study from Iran examined the cost‐effectiveness (Malakouti et al., 2015). A cost‐utility analysis was used. The study found that the costs per‐Quality‐Adjusted Life Year (QALY) gained for GP‐led and nurse‐led groups compared with the control group were 5,740,807 and 5,048,459 Iranian Rial (IRR), respectively. The nurse‐led group's cost was lower by 692,348 IRR (12%), but the significance difference between groups was not analysed by the authors.

### 3.3 Thematic findings

**FIGURE 2:**
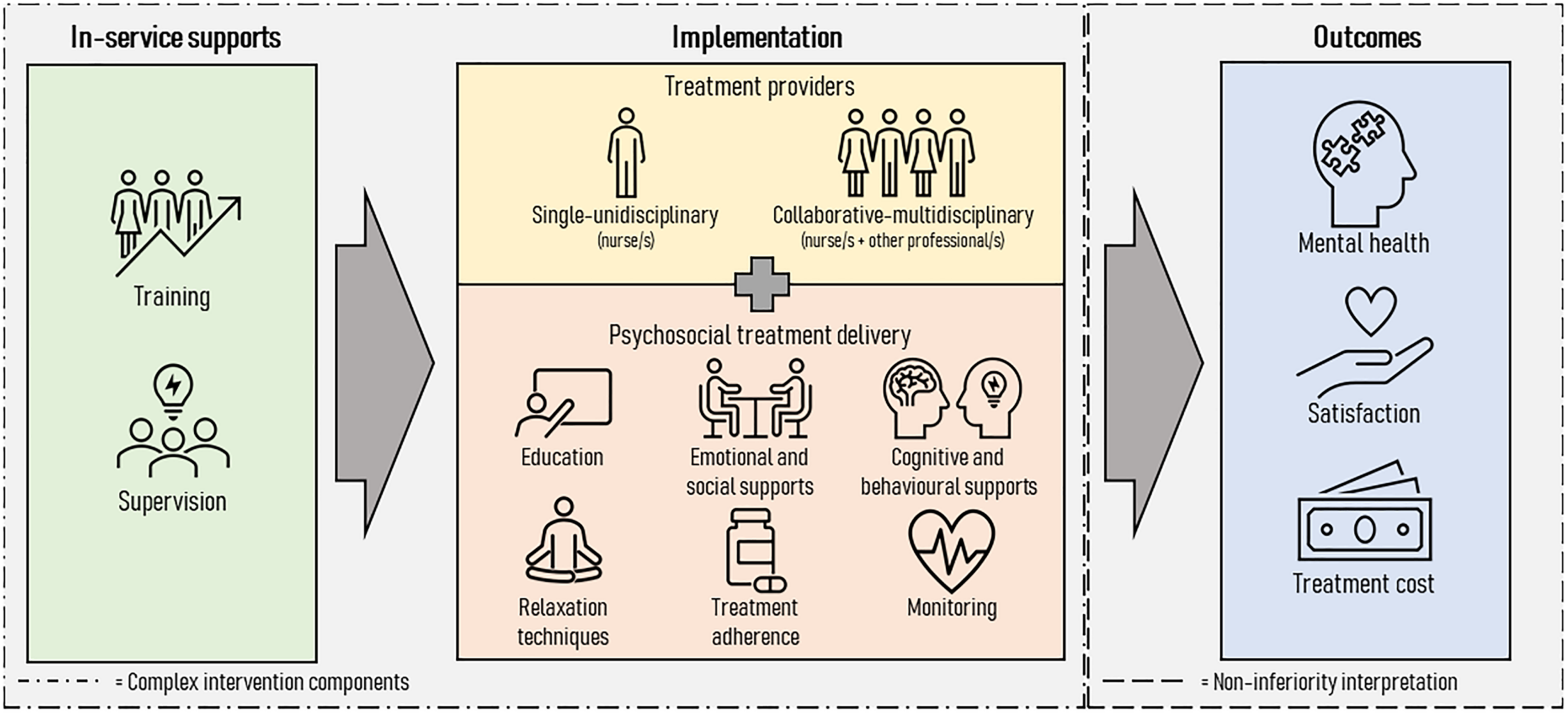
Mental health task‐shifting and task‐sharing theoretical framework

This review has synthesized 10 components of task‐shifting interventions and grouped into three major themes: training and supervision, single and collaborative care and the implementation of psychosocial treatment delivery (Table 3). The theoretical framework of the intervention components is described in Figure 2. The complex task‐shifting intervention includes in‐service supports through training and supervision, two types of treatment providers and some kinds of psychosocial treatments, as well as the outcomes of mental health status, patient satisfaction and cost‐effectiveness.

**TABLE 3:** The themes generated from the analysis

				Implementation								Outcomes		
Themes		In‐service supports		Treatm. provider		Psychosocial treatment delivery								
Study	Complex intervention components	Training	Supervision	Collaborative‐multidisciplinary	Single or unidisciplinary	Education	Emotional and social supports	Cognitive and behavioural changes	Relaxation techniques	Treatment adherence	Monitoring	Mental health improvement	Patient satisfaction	Cost‐effectiveness
Aragonès et al. (2012)*		●	·	●	·	●	·	●	·	●	●	●	·	·
Buszewicz et al. (2016)		●	●	●	·	·	●	●	·	●	●	●	·	·
Casañas et al. (2012)*		●	·	·	●	●	●	●	●	·	·	●	·	·
Dobscha et al. (2006)		●	·	●	·	●	·	·	·	●	●	●	●	·
Ejeby et al. (2014)*		●	●	·	●	●	●	●	●	·	·	●	·	·
Fortney et al. (2013)*		●	●	●	·	●	·	●	·	·	●	●	●	·
Hunkeler et al. (2000)*		●	●	●	·	·	●	·	·	●	●	●	●	·
Malakouti et al. (2015)*		●	●	●	·	●	·	·	·	·	●	●	●	●
Mynors‐Wallis et al. (2000)		●	●	·	●	·	·	●	·	●	·	●	·	·
Rost et al. (2001)*		●	·	●	·	●	·	·	·	·	·	●	●	·
Rost et al. (2002)*		●	·	●	·	·	·	·	·	●	●	●	·	·
Zimmermann et al. (2016)		●	·	●	·	·	·	●	·	·	·	●	·	·
Total		12	6	9	3	7	4	7	2	6	7	12	5	1

* *p* < 0.05.

#### 3.3.1 Training and supervision

All studies involved training for the treatment provider in at least one treatment arm; six studies provided training for all treatment arms. The training duration varies, from one‐point training provided in a couple of hours or days less than a week (*n* = 7) (Aragonès et al., 2012; Buszewicz et al., 2016; Casañas et al., 2012; Dobscha et al., 2006; Fortney et al., 2013; Hunkeler et al., 2000; Malakouti et al., 2015) to serial training with additional coordination sessions (Rost et al., 2001, 2002). Two studies did not disclose the duration but specified the training names: multimodal intervention (Ejeby et al., 2014) and problem‐solving therapy training (Mynors‐Wallis et al., 2000). In one study, the nurses were sent to local primary care centres to directly learn about mental health service (Zimmermann et al., 2016).

Furthermore, six studies provided regular supervision to the treating nurses, with two of these studies also providing additional supervision to the GPs. In Buszewicz et al. (2016), the nurses received a quality assurance visit from a Medical Research Council GP and regular telephone contact from a team of two GPs and one psychologist. Clinical supervision was also undertaken in Fortney et al. (2013) and Hunkeler et al. (2000), mostly by telephone; in Malakouti et al. (2015), the supervision was provided by the principal investigator. In two studies, the supervisors were experts in the respective therapies: the originator of multimodal intervention (Ejeby et al., 2014) and an experienced problem‐solving therapist (Mynors‐Wallis et al., 2000).

#### 3.3.2 Single and collaborative care

In three studies, the intervention arm was led by a single‐unidisciplinary provider, only a nurse. Each patient in two studies received treatment from one nurse (Ejeby et al., 2014; Mynors‐Wallis et al., 2000), while in one study, the patient was treated by two nurses (Casañas et al., 2012). All these studies compared the nurse‐led with the GP‐led intervention. In Casañas et al. (2012), the comparison arm included a nurse as the GP's companion. Meanwhile, Mynors‐Wallis et al. (2000) compared the nurse‐led problem‐solving therapy with three comparators: problem‐solving therapy by doctors, fluvoxamine medication and the combination of both. In nine studies, the nurse actively collaborated with multidisciplinary providers, mainly GP and psychiatrist. Of which, two were exclusively between nurse and GP (Buszewicz et al., 2016; Zimmermann et al., 2016). In five studies, the nurse worked collaboratively with GP and another provider, for example, psychiatrist (Aragonès et al., 2012; Fortney et al., 2013), psychologist, pharmacist (Fortney et al., 2013), peer support volunteer (Hunkeler et al., 2000) or health administrator (Rost et al., 2001, 2002). Two studies showed that the nurse had an exclusive collaboration with a psychiatrist (Dobscha et al., 2006; Malakouti et al., 2015).

Six studies conducted collaboration through shared consultation, in which the nurse case/care manager closely worked and consulted with the other health professionals about the patient's condition (Aragonès et al., 2012; Buszewicz et al., 2016; Dobscha et al., 2006; Hunkeler et al., 2000; Rost et al., 2002; Zimmermann et al., 2016). Two studies involved referral or counter‐referral from the nurses to the other providers (Fortney et al., 2013; Malakouti et al., 2015). Meanwhile, in one study, the nurse and the other providers (GP and administrative staff) worked as a team with distinctive and nonhierarchical roles (Rost et al., 2001).

#### 3.3.3 Psychosocial treatments

The studies indicated that nurses had a role as a psychosocial treatment provider. The treatments consisted of several components, namely, psychoeducation (*n* = 7), emotional and social supports (*n* = 4), cognitive and behavioural change supports (*n* = 7), relaxation techniques (*n* = 2) and treatment adherence supports (*n* = 6). Most cognitive and behavioural changes were delivered through self‐management therapy (Aragonès et al., 2012; Fortney et al., 2013; Zimmermann et al., 2016) or problem‐solving therapy (Buszewicz et al., 2016; Casañas et al., 2012; Mynors‐Wallis et al., 2000); the relaxation techniques were breathing technique (Casañas et al., 2012) and physical training and yoga (Ejeby et al., 2014). Regular monitoring was undertaken in seven studies (Aragonès et al., 2012; Buszewicz et al., 2016; Dobscha et al., 2006; Fortney et al., 2013; Hunkeler et al., 2000; Malakouti et al., 2015; Rost et al., 2002), in which the nurses made contact with the patients and assessed their conditions, including their symptoms, medication, mood and social circumstances.

## 4 DISCUSSION

### 4.1 Non‐inferiority interpretation

The majority of nurse‐led task‐shifting interventions significantly improved mental health symptoms in adults with mental health problems. This result indicates that the nurses can provide mental health intervention normally delivered by GPs, psychiatrists or psychologists. Although few studies indicated that the nurse‐led arm was not superior to the comparator, the outcome demonstrated significant improvement. These studies suggested that the mental health outcomes between nurses and other providers, for example, GPs, psychiatrists and psychologists, were not significantly different. This result means that substituting or redistributing mental health interventions to nurses did not decrease the quality of care. Some systematic reviews concerning various health issues indicated that interventions shifted to or shared with nurses were comparable with interventions delivered by health providers with higher qualifications. These interventions could reduce the treatment cost and address health‐care workforce shortage without compromising patient health outcomes (Callaghan et al., 2010; Lassi et al., 2013; Martínez‐González et al., 2015; Mdege et al., 2013; Ogedegbe et al., 2014; Penazzato et al., 2014).

Decreased quality is an expected expense for interventions shifted to or shared with the less qualified providers. Accordingly, many studies demonstrated what we call ‘noninferiority interpretation’, which means that the ‘target’ provider (i.e. to which the tasks are shifted or shared) does not necessarily have to be superior to the ‘original’ provider (i.e. from which the tasks originally come) to call an intervention successful and worthy of being implemented. Therefore, it only requires the target health providers to be at least not worse than the original as the purpose of task‐shifting research corresponds with the noninferiority trial rationale, that is, to find the ‘good substitute’ of medical or health treatment (Mauri & D'Agostino, 2017).

### 4.2 Complex intervention

Shifting and sharing mental health‐care delivery to the nurses requires the involvement of several independent and interdependent components; accordingly, this intervention can be considered as a complex intervention (Campbell et al., 2000). The review has synthesized 10 components of the complex intervention, which were grouped into three major themes: in‐service supports, treatment provider and psychosocial treatment delivery. These themes build up the conceptual framework of task‐shifting complex interventions involving the nurses in mental health context (see Figure 2).

The nurse‐delivered task‐shifting intervention usually started with the provision of training and supervision to the nurses to whom the tasks are being shifted or shared. These supports are usually provided preceding the services, though some sessions can be conducted during the services, particularly the supervisions. These two components are intended to enhance the nurse capabilities in undertaking some competencies that are beyond their basic qualification. Regardless of the purpose and delivery method, some evidence concerning various health issues showed that training and supervision are the inevitable parts of task‐shifting that have many benefits (Federspiel et al., 2018; Hoeft et al., 2018; Raviola et al., 2019). Despite the benefits, most evidence revealed that training and supervision did not directly improve patient clinical outcomes. Both are supporting components which improve the providers' capabilities but do not have an indirect effect on patient outcomes.

As mentioned before, there are differences between task‐shifting and task‐sharing. The involvement of single‐provider or collaboration can distinguish whether an intervention is shifted or shared, respectively (Orkin et al., 2021). In this review, collaborative care studies outnumbered single‐provider care studies, nine compared with three studies, respectively. Task‐sharing intervention was more frequently studied than task‐shifting. However, this review was unable to determine which approach producing better results as the percentage of studies with significant results is the same (66.7%). Moreover, the evidence that directly compares task‐shifting and task‐sharing are still limited. When discussed separately, both task‐shifting and task‐sharing have considerable benefits. However, evidence‐based collaborative care for depression and anxiety was not widely studied; therefore, its broader applicability requires further scrutiny.

### 4.3 Review limitations

Despite the positive results found in most evidence, the effect estimates of the studies should be interpreted carefully. First, the internal validity in most studies was ambiguous due to the high RoB. The nature of psychosocial interventions means that some biases cannot be avoided. For instance, it is impossible to blind both the participants and treatment providers, making them aware of the treatment allocation as the interventions were delivered face‐to‐face. Second, the external validity was limited because almost all studies were conducted in European and North American high‐income countries. This means that Asian, African, Oceanian and South American regions as well as the low‐ and middle‐income countries were still underrepresented.

Furthermore, this systematic review did not register and publish a prespecified protocol before the review starts due to time constraint and limited resources. The review only included studies written in English. There is a possibility that studies written in languages other than English may meet the inclusion and exclusion criteria. However, these studies may not be adequately screened due to the reviewer's language barrier. Finally, none of the studies included were explicitly claimed as task‐shifting/sharing research. The eligibility criteria were based on health providers involvement which may resemble a task‐shifting situation. This methodological choice potentially undermines the internal validity of the review.

## 5 CONCLUSIONS

The practical implication of this study is that the government could employ nurses to deliver mental health interventions in the absence of mental health services in primary care due to the shortage of mental health specialists. However, it should be noted that appropriate training and supervision are required before the nurses are ready to undertake the new roles in practice. Expanding the nurses' scope of practice may require regulatory changes to ensure that substituting mental health interventions to nurses are addressed by the laws (Benton et al., 2020). Besides, the task‐shifting and task‐sharing interventions require a well‐planned and gradual implementation strategy; an outright implementation may instantly overcome the treatment gap, but it could induce burnout and workplace‐related distress (Padmanathan & De Silva, 2013).

There is an agenda that can be developed in further studies: first, to compare the effect of task‐shifting and task‐sharing interventions, as well as to identify which approach is more recommended in a particular context; second, to calculate the cost‐effectiveness of the nurse‐led task‐shifting interventions; and third, to identify the impact of technology on task‐shifting implementation.

## CONFLICT OF INTEREST

The authors declare no conflict of interest.

## AUTHORSHIP STATEMENT

GEA and IW designed the study. GEA collected and analysed the data. GEA and IW prepared the manuscript. All authors approved the final version for submission.

## Supporting information




**Data S1.** Supporting Information

## Data Availability

The data that support the findings of this study are available from the corresponding author upon reasonable request.

## APPENDIX A

**TABLE A1:** Various mental health outcomes measurements

Study	PHQ‐9	SF‐12	SF‐36	BDI (II)	WSAS	EQ‐5D	EQ‐VAS	CIDI	HSCL‐20	CPRS	mCES‐D	GSES	GHQ‐28	HDRS	Antidepressant use	QALYs	Satisfaction	Others
Aragonès et al. (2012)	●	●	·	·	·	·	·	·	·	·	·	·	·	·	·	·	·	·
Buszewicz et al. (2016)	·	·	·	●	●	·	●	●	·	·	·	·	·	·	●	·	·	·
Casañas et al. (2012)	·	·	·	●^a^	·	●	·	·	·	·	·	·	·	·	·	·	·	·
Dobscha et al. (2006)	●	·	●	·	·	·	·	·	●	·	·	·	·	·	●	·	●	·
Ejeby et al. (2014)	·	·	●	·	·	·	·	·	·	●	·	·	·	·	·	·	·	●
Fortney et al. (2013)	·	●	·	·	·	·	·	·	●^b^	·	·	·	·	·	·	·	●	●
Hunkeler et al. (2000)	·	●	·	●^b^	·	·	·	·	·	·	·	·		●^b^	·	·	●	·
Malakouti et al. (2015)	·	·	·	·	·	·	·	·	·	·	·	·	●	·	·	●	●	●
Mynors‐Wallis et al. (2000)	·	·	·	●	·	·	·	·	·	·	·	·	·	●	·	·	·	●
Rost et al. (2001)	·	·	·	·	·	·	·	·	·	·	●^b^	·	·	·	·	·	●	·
Rost et al. (2002)	·	·	●	·	·	·	·	·	·	·	●^a^	·	·	·	·	·	·	·
Zimmermann et al. (2016)	●	·	·	·	·	●	·	·	·	·	·	●	·	·	·	·	·	·
**Total**	**3x**	**3x**	**3x**	**4x**	**1x**	**2x**	**1x**	**1x**	**2x**	**1x**	**2x**	**1x**	**1x**	**2x**	**2x**	**1x**	**5x**	**NA**

Abbreviations: BDI (II), Beck Depression Inventory (Version II); CIDI, WHO Composite International Diagnostic Interview; CPRS, Comprehensive Psychopathological EQ‐5D, 5‐item EuroQOL Depression; Rating Scale; EQ‐VAS, EuroQOL Visual Analogue Scale; GHQ‐28, 28‐item General Health Questionnaire (GHQ‐28); GSES, General Self‐Efficacy Scale; HDRS, Hamilton Depression Rating Scale; HSCL‐20, 20‐item Hopkins Symptom Checklist; mCES‐D, modified version of Centre for Epidemiologic Studies—Depression Scale; PHQ‐9, 9‐item Patient Health Questionnaire; QALYs, Quality‐Adjusted Life Years; SF‐12, 12‐item Short Form Survey; SF‐36, 36‐item Short Form Survey; WSAS, Work and Social Activity Scale.

^a^ Remission.

^b^ Response.

## References

[ijn13046-bib-0001] Afolabi, O. , Abboah‐Offei, M. , Nkhoma, K. , & Evans, C. (2019). Task‐shifting must recognise the professional role of nurses. The Lancet Global Health, 7(10), e1328–e1329. 10.1016/S2214-109X(19)30358-4 31537365

[ijn13046-bib-0002] Alonso, J. , Liu, Z. , Evans‐Lacko, S. , Sadikova, E. , Sampson, N. , Chatterji, S. , Abdulmalik, J. , Aguilar‐Gaxiola, S. , Al‐Hamzawi, A. , Andrade, L. H. , Bruffaerts, R. , Cardoso, G. , Cia, A. , Florescu, S. , de Girolamo, G. , Gureje, O. , Haro, J. M. , He, Y. , de Jonge, P. , … Thornicroft, G. (2018). Treatment gap for anxiety disorders is global: Results of the World Mental Health Surveys in 21 countries. Depression and Anxiety, 35(3), 195–208. 10.1002/da.22711 29356216 PMC6008788

[ijn13046-bib-0003] Aragonès, E. , Lluís Piñol, J. , Caballero, A. , López‐Cortacans, G. , Casaus, P. , Maria Hernández, J. , Badia, W. , & Folch, S. (2012). Effectiveness of a multi‐component programme for managing depression in primary care: A cluster randomized trial. The INDI project. Journal of Affective Disorders, 142(1–3), 297–305. 10.1016/j.jad.2012.05.020 23062747

[ijn13046-bib-0004] Benton, D. C. , Ferguson, S. L. , & Holloway, A. (2020). Task shifting: A high‐level analysis of scholarship. Journal of Nursing Regulation, 11(2), 4–11. 10.1016/S2155-8256(20)30104-6

[ijn13046-bib-0005] Bruckner, T. A. , Scheffler, R. M. , Shen, G. , Yoon, J. , Chisholm, D. , Morris, J. , Fulton, B. D. , dal Poz, M. R. , & Shekhar, S. (2011). The mental health workforce gap in low‐ and middle‐income countries: A needs‐based approach. Bulletin of the World Health Organization, 89(3), 184–194. 10.2471/BLT.10.082784 21379414 PMC3044251

[ijn13046-bib-0006] Buszewicz, M. , Griffin, M. , McMahon, E. M. , Walters, K. , & King, M. (2016). Practice nurse‐led proactive care for chronic depression in primary care: A randomised controlled trial. British Journal of Psychiatry, 208(4), 374–380. 10.1192/bjp.bp.114.153312 26795423

[ijn13046-bib-0007] Butryn, B. , Marchionni, L. , & Sholevar, F. (2017). The shortage of psychiatrists and other mental health providers: Causes, current state, and potential solutions. International Journal of Academic Medicine, 3(1), 5. 10.4103/IJAM.IJAM_49_17

[ijn13046-bib-0008] Callaghan, M. , Ford, N. , & Schneider, H. (2010). A systematic review of task‐shifting for HIV treatment and care in Africa. Human Resources for Health, 8(8). 10.1186/1478-4491-8-8 PMC2873343 20356363

[ijn13046-bib-0009] Campbell, M. , Fitzpatrick, R. , Haines, A. , Kinmonth, A. L. , Sandercock, P. , Spiegelhalter, D. , & Tyrer, P. (2000). Framework for design and evaluation of complex interventions to improve health. British Medical Journal, 321(7262), 694–696. 10.1136/bmj.321.7262.694 10987780 PMC1118564

[ijn13046-bib-0010] Casañas, R. , Catalán, R. , del Val, J. L. , Real, J. , Valero, S. , & Casas, M. (2012). Effectiveness of a psycho‐educational group program for major depression in primary care: A randomized controlled trial. BMC Psychiatry, 12(1), 230. 10.1186/1471-244X-12-230 23249399 PMC3551665

[ijn13046-bib-0011] Dham, P. , Colman, S. , Saperson, K. , McAiney, C. , Lourenco, L. , Kates, N. , & Rajji, T. K. (2017). Collaborative care for psychiatric disorders in older adults: A systematic review. The Canadian Journal of Psychiatry, 62(11), 761–771. 10.1177/0706743717720869 28718325 PMC5697628

[ijn13046-bib-0012] Dobscha, S. K. , Corson, K. , Hickam, D. H. , Perrin, N. A. , Kraemer, D. F. , & Gerrity, M. S. (2006). Depression decision support in primary care: A cluster randomized trial. Annals of Internal Medicine, 145(7), 477–487. 10.7326/0003-4819-145-7-200610030-00005 17015865

[ijn13046-bib-0013] Ejeby, K. , Savitskij, R. , Öst, L. G. , Ekbom, A. , Brandt, L. , Ramnerö, J. , Åsberg, M. , & Backlund, L. G. (2014). Randomized controlled trial of transdiagnostic group treatments for primary care patients with common mental disorders. Family Practice, 31(3), 273–280. 10.1093/fampra/cmu006 24642702 PMC4024961

[ijn13046-bib-0014] Ekers, D. , Murphy, R. , Archer, J. , Ebenezer, C. , Kemp, D. , & Gilbody, S. (2013). Nurse‐delivered collaborative care for depression and long‐term physical conditions: A systematic review and meta‐analysis. Journal of Affective Disorders, 149(1–3), 14–22. 10.1016/j.jad.2013.02.032 23545062

[ijn13046-bib-0015] Evans‐Lacko, S. , Aguilar‐Gaxiola, S. , Al‐Hamzawi, A. , Alonso, J. , Benjet, C. , Bruffaerts, R. , Chiu, W. T. , Florescu, S. , De Girolamo, G. , Gureje, O. , Haro, J. M. , He, Y. , Hu, C. , Karam, E. G. , Kawakami, N. , Lee, S. , Lund, C. , Kovess‐Masfety, V. , Levinson, D. , … Wojtyniak, B. (2018). Socio‐economic variations in the mental health treatment gap for people with anxiety, mood, and substance use disorders: Results from the WHO World Mental Health (WMH) surveys. Psychological Medicine, 48(9), 1560–1571. 10.1017/S0033291717003336 29173244 PMC6878971

[ijn13046-bib-0016] Federspiel, F. , Mukhopadhyay, S. , Milsom, P. J. , Scott, J. W. , Riesel, J. N. , & Meara, J. G. (2018). Global surgical, obstetric, and anesthetic task shifting: A systematic literature review. Surgery, 164(3), 553–558. 10.1016/j.surg.2018.04.024 30145999

[ijn13046-bib-0017] Fortney, J. C. , Pyne, J. M. , Mouden, S. B. , Mittal, D. , Hudson, T. J. , Schroeder, G. W. , Williams, D. K. , Bynum, C. A. , Mattox, R. , & Rost, K. M. (2013). Practice‐based versus telemedicine‐based collaborative care for depression in rural federally qualified health centers: A pragmatic randomized comparative effectiveness trial. American Journal of Psychiatry, 170(4), 414–425. 10.1176/appi.ajp.2012.12050696 23429924 PMC3816374

[ijn13046-bib-0018] Galvin, M. , & Byansi, W. (2020). A systematic review of task shifting for mental health in Sub‐Saharan Africa. International Journal of Mental Health, 49, 1–25. 10.1080/00207411.2020.1798720

[ijn13046-bib-0019] Gureje, O. , Oladeji, B. D. , Montgomery, A. A. , Bello, T. , Kola, L. , Ojagbemi, A. , Chisholm, D. , & Araya, R. (2019). Effect of a stepped‐care intervention delivered by lay health workers on major depressive disorder among primary care patients in Nigeria (STEPCARE): A cluster‐randomised controlled trial. The Lancet Global Health, 7(7), e951–e960. 10.1016/S2214-109X(19)30148-2 31097414 PMC6559947

[ijn13046-bib-0020] Halcomb, E. J. , McInnes, S. , Patterson, C. , & Moxham, L. (2018). Nurse‐delivered interventions for mental health in primary care: A systematic review of randomized controlled trials. Family Practice, 36(1), 64–71. 10.1093/fampra/cmy101 30364968

[ijn13046-bib-0021] Hay, S. I. , Abajobir, A. A. , Abate, K. H. , Abbafati, C. , Abbas, K. M. , Abd‐Allah, F. , Abdulkader, R. S. , Abdulle, A. M. , Abebo, T. A. , Abera, S. F. , Aboyans, V. , Abu‐Raddad, L. J. , Ackerman, I. N. , Adedeji, I. A. , Adetokunboh, O. , Afshin, A. , Aggarwal, R. , Agrawal, S. , Agrawal, A. , … Murray, C. J. L. (2017). Global, regional, and national disability‐adjusted life‐years (DALYs) for 333 diseases and injuries and healthy life expectancy (HALE) for 195 countries and territories, 1990–2016: a systematic analysis for the Global Burden of Disease Study 2016. The Lancet, 390(10100), 1260–1344. 10.1016/S0140-6736(17)32130-X PMC5605707 28919118

[ijn13046-bib-0022] Higgins, J. , Thomas, J. , Chandler, J. , Cumpston, M. , Li, T. , Page, M. , & Welch, V. (2019). Cochrane handbook for systematic reviews of interventions version 6.0 (updated July 2019). In J. Higgins , J. Thomas , J. Chandler , M. Cumpston , T. Li , M. Page , & V. Welch (Eds.), Cochrane. http://www.training.cochrane.org/handbook, 10.1002/9781119536604

[ijn13046-bib-0023] Ho, F. Y. Y. , Yeung, W. F. , Ng, T. H. Y. , & Chan, C. S. (2016). The efficacy and cost‐effectiveness of stepped care prevention and treatment for depressive and/or anxiety disorders: A systematic review and meta‐analysis. Scientific Reports, 6(1), 29281. 10.1038/srep29281 27377429 PMC4932532

[ijn13046-bib-0024] Hoeft, T. J. , Fortney, J. C. , Patel, V. , & Unützer, J. (2018). Task‐sharing approaches to improve mental health care in rural and other low‐resource settings: A systematic review. Journal of Rural Health, 34(1), 48–62. 10.1111/jrh.12229 PMC5509535 28084667

[ijn13046-bib-0025] Hunkeler, E. M. , Meresman, J. F. , Hargreaves, W. A. , Fireman, B. , Berman, W. H. , Kirsch, A. J. , Groebe, J. , Hurt, S. W. , Braden, P. , Getzell, M. , Feigenbaum, P. A. , Peng, T. , & Salzer, M. (2000). Efficacy of nurse telehealth care and peer support in augmenting treatment of depression in primary care. Archives of Family Medicine, 9(8), 700–708. 10.1001/archfami.9.8.700 10927707

[ijn13046-bib-0026] Kazdin, A. E. (2017). Addressing the treatment gap: A key challenge for extending evidence‐based psychosocial interventions. Behaviour Research and Therapy, 88, 7–18. 10.1016/j.brat.2016.06.004 28110678

[ijn13046-bib-0027] Lassi, Z. S. , Cometto, G. , Huicho, L. , & Bhutta, Z. A. (2013). Quality of care provided by mid‐level health workers: systematic review and meta‐analysis. Bulletin of the World Health Organization, 91(11), 824–833I. 10.2471/BLT.13.118786 24347706 PMC3853954

[ijn13046-bib-0028] Malakouti, S. K. , Mirabzadeh, A. , Nojomi, M. , Tonkaboni, A. A. , Nadarkhani, F. , Mirzaie, M. , & Chimeh, N. (2015). Clinical outcomes and cost effectiveness of two aftercare models provided by general physicians and nurses to patients with severe mental illness. Medical Journal of the Islamic Republic of Iran, 29, 196. 26157714 PMC4476229

[ijn13046-bib-0029] Martínez‐González, N. A. , Tandjung, R. , Djalali, S. , & Rosemann, T. (2015). The impact of physician–nurse task shifting in primary care on the course of disease: A systematic review. Human Resources for Health, 13(1), 55. 10.1186/s12960-015-0049-8 26149447 PMC4493821

[ijn13046-bib-0030] Mauri, L. , & D'Agostino, R. B. (2017). Challenges in the design and interpretation of noninferiority trials. New England Journal of Medicine, 377(14), 1357–1367. 10.1056/NEJMra1510063 28976859

[ijn13046-bib-0031] Mays, N. , Pope, C. , & Popay, J. (2005). Systematically reviewing qualitative and quantitative evidence to inform management and policy‐making in the health field. Journal of Health Services Research and Policy, 10(SUPPL. 6), 6–20. 10.1258/1355819054308576 16053580

[ijn13046-bib-0032] Mdege, N. D. , Chindove, S. , & Ali, S. (2013). The effectiveness and cost implications of task‐shifting in the delivery of antiretroviral therapy to HIV‐infected patients: a systematic review. Health Policy and Planning, 28(3), 223–236. 10.1093/heapol/czs058 22738755

[ijn13046-bib-0033] Moher, D. , Liberati, A. , Tetzlaff, J. , & Altman, D. G. (2009). Preferred reporting items for systematic reviews and meta‐analyses: The PRISMA statement. PLoS Medicine, 6(7), e1000097. 10.1371/journal.pmed.1000097 19621072 PMC2707599

[ijn13046-bib-0034] Mynors‐Wallis, L. M. , Gath, D. H. , Day, A. , & Baker, F. (2000). Randomised controlled trial of problem solving treatment, antidepressant medication, and combined treatment for major depression in primary care. British Medical Journal, 320(7226), 26–30. 10.1136/bmj.320.7226.26 10617523 PMC27250

[ijn13046-bib-0035] Ogedegbe, G. , Gyamfi, J. , Plange‐Rhule, J. , Surkis, A. , Rosenthal, D. M. , Airhihenbuwa, C. , Iwelunmor, J. , & Cooper, R. (2014). Task shifting interventions for cardiovascular risk reduction in low‐income and middle‐income countries: a systematic review of randomised controlled trials. BMJ Open, 4(10), e005983. 10.1136/bmjopen-2014-005983 PMC4202019 25324324

[ijn13046-bib-0036] Ola, B. A. , & Atilola, O. (2019). Task‐shifted interventions for depression delivered by lay primary health‐care workers in low‐income and middle‐income countries. The Lancet Global Health, 7(7), e829–e830. 10.1016/S2214-109X(19)30197-4 31097415

[ijn13046-bib-0037] Orkin, A. M. , Rao, S. , Venugopal, J. , Kithulegoda, N. , Wegier, P. , Ritchie, S. D. , VanderBurgh, D. , Martiniuk, A. , Salamanca‐Buentello, F. , & Upshur, R. (2021). Conceptual framework for task shifting and task sharing: an international Delphi study. Human Resources for Health, 19(1), 1–8. 10.1186/s12960-021-00605-z 33941191 PMC8091141

[ijn13046-bib-0038] Padmanathan, P. , & De Silva, M. J. (2013). The acceptability and feasibility of task‐sharing for mental healthcare in low and middle income countries: A systematic review. Social Science and Medicine, 97, 82–86. 10.1016/j.socscimed.2013.08.004 24161092

[ijn13046-bib-0039] Patel, V. , Chowdhary, N. , Rahman, A. , & Verdeli, H. (2011). Improving access to psychological treatments: Lessons from developing countries. Behaviour Research and Therapy, 49(9), 523–528. 10.1016/j.brat.2011.06.012 21788012 PMC3242164

[ijn13046-bib-0040] Patel, V. , Maj, M. , Flisher, A. J. , De Silva, M. J. , Koschorke, M. , Prince, M. , Tempier, R. , Riba, M. B. , Sanchez, M. , Campodonico, F. D. , Risco, L. , Gask, L. , Wahlberg, H. , Roca, M. , Lecic‐Tosevski, D. , Soghoyan, A. , Moussaoui, D. , Baddoura, C. , Adeyemi, J. , … Richardson, G. (2010). Reducing the treatment gap for mental disorders: A WPA survey. World Psychiatry, 9(3), 169–176. 10.1002/j.2051-5545.2010.tb00305.x 20975864 PMC2953637

[ijn13046-bib-0055] Patel, V. , Saxena, S. , Lund, C. , Thornicroft, G. , Baingana, F. , Bolton, P. , Chisholm, D. , Collins, P. Y. , Cooper, J. L. , Eaton, J. , Herrman, H. , Herzallah, M. M. , Huang, Y. , Jordans, M. J. D. , Kleinman, A. , Medina‐Mora, M. E. , Morgan, E. , Niaz, U. , Omigbodun, O. , … UnÜtzer, J. (2018). The lancet commission on global mental health and sustainable development. The Lancet, 392(10157), 1553–1598. 10.1016/s0140-6736(18)31612-x 30314863

[ijn13046-bib-0041] Penazzato, M. , Davies, M.‐A. , Apollo, T. , Negussie, E. , & Ford, N. (2014). Task shifting for the delivery of pediatric antiretroviral treatment. JAIDS Journal of Acquired Immune Deficiency Syndromes, 65(4), 414–422. 10.1097/QAI.0000000000000024 24583614

[ijn13046-bib-0042] Raviola, G. , Naslund, J. A. , Smith, S. L. , & Patel, V. (2019). Innovative models in mental health delivery systems: Task sharing care with non‐specialist providers to close the mental Health treatment gap. Current Psychiatry Reports, 21(6), 44. 10.1007/s11920-019-1028-x 31041554

[ijn13046-bib-0043] Rebello, T. J. , Marques, A. , Gureje, O. , & Pike, K. M. (2014). Innovative strategies for closing the mental health treatment gap globally. Current Opinion in Psychiatry, 27(4), 308–314. 10.1097/YCO.0000000000000068 24840160

[ijn13046-bib-0044] Rost, K. , Nutting, P. , Smith, J. , Werner, J. , & Duan, N. (2001). Improving depression outcomes in community primary care practice: A randomized trial of the QuEST intervention. Journal of General Internal Medicine, 16(3), 143–149. 10.1111/j.1525-1497.2001.00537.x 11318908 PMC1495192

[ijn13046-bib-0045] Rost, K. , Nutting, P. , Smith, J. L. , Elliott, C. E. , & Dickinson, M. (2002). Managing depression as a chronic disease: a randomised trial of ongoing treatment in primary care. BMJ, 325(7370), 934. 10.1136/bmj.325.7370.934 12399343 PMC130058

[ijn13046-bib-0046] Shahmalak, U. , Blakemore, A. , Waheed, M. W. , & Waheed, W. (2019). The experiences of lay health workers trained in task‐shifting psychological interventions: a qualitative systematic review. International Journal of Mental Health Systems, 13(1), 64. 10.1186/s13033-019-0320-9 31636699 PMC6790996

[ijn13046-bib-0047] Sterne, J. A. C. , Savović, J. , Page, M. J. , Elbers, R. G. , Blencowe, N. S. , Boutron, I. , Cates, C. J. , Cheng, H.‐Y. , Corbett, M. S. , Eldridge, S. M. , Emberson, J. R. , Hernán, M. A. , Hopewell, S. , Hróbjartsson, A. , Junqueira, D. R. , Jüni, P. , Kirkham, J. J. , Lasserson, T. , Li, T. , … Higgins, J. P. T. (2019). RoB 2: a revised tool for assessing risk of bias in randomised trials. BMJ, l4898.. 10.1136/bmj.l4898 31462531

[ijn13046-bib-0048] Thornicroft, G. (2007). Most people with mental illness are not treated. The Lancet, 370(9590), 807–808. 10.1016/S0140-6736(07)61392-0 17826153

[ijn13046-bib-0049] van Ginneken, N. , Tharyan, P. , Lewin, S. , Rao, G. N. , Meera, S. , Pian, J. , Chandrashekar, S. , & Patel, V. (2013). Non‐specialist health worker interventions for the care of mental, neurological and substance‐abuse disorders in low‐ and middle‐income countries. Cochrane Database of Systematic Reviews, *2013*(11), 131–132. 10.1002/14651858.CD009149.pub2 24249541

[ijn13046-bib-0050] van Straten, A. , Hill, J. , Richards, D. A. , & Cuijpers, P. (2015). Stepped care treatment delivery for depression: a systematic review and meta‐analysis. Psychological Medicine, 45(2), 231–246. 10.1017/S0033291714000701 25065653

[ijn13046-bib-0051] Verhey, I. J. , Ryan, G. K. , Scherer, N. , & Magidson, J. F. (2020). Implementation outcomes of cognitive behavioural therapy delivered by non‐specialists for common mental disorders and substance‐use disorders in low‐ And middle‐income countries: A systematic review. International Journal of Mental Health Systems, 14(1), 1–14. 10.1186/s13033-020-00372-9 32514304 PMC7260765

[ijn13046-bib-0052] WHO . (2008). Task shifting: Global recomendations and guidelines.

[ijn13046-bib-0053] Zimmermann, T. , Puschmann, E. , van den Bussche, H. , Wiese, B. , Ernst, A. , Porzelt, S. , Daubmann, A. , & Scherer, M. (2016). Collaborative nurse‐led self‐management support for primary care patients with anxiety, depressive or somatic symptoms: Cluster‐randomised controlled trial (findings of the SMADS study). International Journal of Nursing Studies, 63, 101–111. 10.1016/j.ijnurstu.2016.08.007 27611093

