# Supplementary material for: Nurse‐led task‐shifting strategies to substitute for mental health specialists in primary care: A systematic review
Source: Int J Nurs Pract. 2022 Mar 13;28(5):e13046. doi: 10.1111/ijn.13046 (PMC9786659; doi:10.1111/ijn.13046)
Supplement: Supplementary file 1 — Data S1. Supporting Information [file IJN-28-e13046-s001.pdf]

SUPPLEMENTARY 1

Search terms, platform and databases used in the review

Platform	Database(s)	BOOLEAN search formula
EBSCOhost	CINAHL; MEDLINE	(Primary health care/ or primary care or community care or community-based* or community-dwelling) AND AB (Nursing/ or nurses/ or lay health worker* or non-specialist health worker* or NSHW* or allied health personnel/ or task shifting or task sharing or personnel staffing or task substitution or treatment gap or unmet need or mhGAP or nurse-led or nurse-delivered) AND (Mental health/ or mental disorders/ or anxiety disorders/ or mood disorders/ or bipolar and related disorders/ or depression/ or panic disorder/ or obsessive-compulsive disorder/ or phobic disorder/ or depressive disorder/ or post-traumatic stress disorders/ or psychiatric condition* or mental illness* or PTSD or psychological*) AND AB (Clinical trial/ or controlled clinical trial/ or randomized controlled trial/ or randomised controlled trial* or RCT*)
OVID	EBM Reviews – Cochrane Database of Systematic Reviews; Ovid MEDLINE(R); APA PsycInfo; EMBASE	(Nursing or nurse* or lay health worker* or non-specialist health worker* or NSHW* or allied health personnel or task shifting or task sharing or personnel staffing or task substitution or treatment gap or unmet need or mhGAP or nurse-led or nurse-delivered).ab. and (Mental health or mental disorder* or anxiety* or mood disorder* or bipolar* or depression or panic* or obsessive-compulsive disorder* or phobi* or depressive disorder* or post-traumatic stress disorder* or psychiatric condition* or mental illness* or PTSD or psychological*).af. and

Platform	Database(s)	BOOLEAN search formula
		(Primary health care or primary care or community care or community-based* or community-dwelling).af. and (Clinical trial* or controlled clinical trial* or randomized controlled trial* or randomised controlled trial* or RCT*).ab.
PubMed	PubMed	(((Nursing[Title/Abstract] OR nurse*[Title/Abstract] OR lay health worker*[Title/Abstract] OR non-specialist health worker*[Title/Abstract] OR NSHW*[Title/Abstract] OR allied health personnel[Title/Abstract] OR task shifting[Title/Abstract] OR task sharing[Title/Abstract] OR personnel staffing[Title/Abstract] OR task substitution[Title/Abstract] OR treatment gap[Title/Abstract] OR unmet need[Title/Abstract] OR mhGAP[Title/Abstract] OR nurse-led[Title/Abstract] OR nurse-delivered[Title/Abstract]) AND (Mental health or mental disorder* or anxiety* or mood disorder* or bipolar* or depression or panic* or obsessive-compulsive disorder* or phobi* or depressive disorder* or post-traumatic stress disorder* or psychiatric condition* or mental illness* or PTSD or psychological*)) AND (Primary health care or primary care or community care or community-based* or community-dwelling)) AND (Clinical trial*[Title/Abstract] OR controlled clinical trial*[Title/Abstract] OR randomized controlled trial*[Title/Abstract] OR randomised controlled trial*[Title/Abstract] OR RCT*[Title/Abstract])
Web of Science	Web of Science Core Collection	TOPIC: (Nursing or nurse* or lay health worker* or non-specialist health worker* or NSHW* or allied health personnel or task shifting or task sharing or personnel staffing or task substitution or treatment gap or unmet need or mhGAP or

Platform	Database(s)	BOOLEAN search formula
		nurse-led or nurse-delivered) AND TOPIC: (Mental health or mental disorder* or anxiety* or mood disorder* or bipolar* or depression or panic* or obsessive-compulsive disorder* or phobi* or depressive disorder* or post-traumatic stress disorder* or psychiatric condition* or mental illness* or PTSD or psychological*) AND TOPIC: (Primary health care or primary care or community care or community-based* or community-dwelling) AND TOPIC: (Clinical trial* or controlled clinical trial* or randomized controlled trial* or randomised controlled trial* or RCT*)
ProQuest	Dissertation and Thesis	(Primary health care or primary care or community care or community-based* or community-dwelling) AND ab(Nursing or nurses or lay health worker* or non-specialist health worker* or NSHW* or allied health personnel or task shifting or task sharing or personnel staffing or task substitution or treatment gap or unmet need or mhGAP or nurse-led or nurse-delivered) AND (Mental health or mental disorders or anxiety disorders or mood disorders or bipolar and related disorders or depression or panic disorder or obsessive-compulsive disorder or phobic disorder or depressive disorder or post-traumatic stress disorders or psychiatric condition* or mental illness* or PTSD or psychological*) AND ab(Clinical trial or controlled clinical trial or randomized controlled trial or randomised controlled trial* or RCT*)
